# An Unusual Case of Clear Cell Chondrosarcoma with Very Late Recurrence and Lung Metastases, 29 Years after Primary Surgery

**DOI:** 10.1155/2014/109569

**Published:** 2014-07-20

**Authors:** Minna Laitinen, Jyrki Nieminen, Toni-Karri Pakarinen

**Affiliations:** ^1^Department of Orthopaedics and Traumatology, Unit of Musculoskeletal Surgery, Tampere University Hospital, P.O. Box 2000, 33521 Tampere, Finland; ^2^Coxa Hospital for Joint Replacement, Biokatu 6, 33520 Tampere, Finland

## Abstract

Clear cell chondrosarcoma is a rare bone neoplasm with low-grade clinical course and the potential to metastasize to the skeleton and lungs. The aim of this report is to present a case that is extremely rare, but in accordance with the literature where the clear cell chondrosarcoma reportedly has a tendency for late metastases. In our patient the primary surgery was intralesional, since it was mistakenly interpreted as a benign tumour in the early 80s. The local recurrence and lung metastases occurred, however, 29 years after the initial treatment. The local recurrence was resected with wide margins, no additional surgery or oncological treatments were given, and two and half years postoperatively patient is doing well and there is no progression in the disease. In conclusion, it is important to have a long follow-up to the clear cell chondrosarcoma patients even for decades or lifelong, because the malignancy tends to metastasize or recur after an extended period. The course of metastasized disease may be unusually slow, so relatively aggressive treatment in metastasized and recurring cases is justified.

## 1. Introduction

Clear cell chondrosarcoma (CCCS) is a rare low-grade malignant tumour of the bone comprising about 1.6%–5.4% of all chondrosarcomas [1, 2]. It is an uncommon variant of chondrosarcoma and was first described by Unni et al. in 1976 [3] and since then more than two hundred cases have been reported in the literature. Before the diagnosis was set, the tumour was often mistakenly characterized as benign. Despite being low malignant the tumour has metastatic potential and overall mortality of 15% [2–5]. Recurrences are not uncommon even in cases with wide resection and metastases may occur after intralesional or wide resection [1, 2, 6].

We report an extremely unusual case of CCCS where the local recurrence and nonsymptomatic lung metastases occurred 29 years after the primary surgery. We also reviewed 41 articles written in English of which 22 were case reports [7–29] and 13 reports with limited clinical data [4, 7, 9, 20, 30–40]. Six articles had more than five cases and clinical data and these reports were studied more carefully [1–3, 5, 6, 41] (Table 1).

## 2. Case Presentation

**Figure 1:**
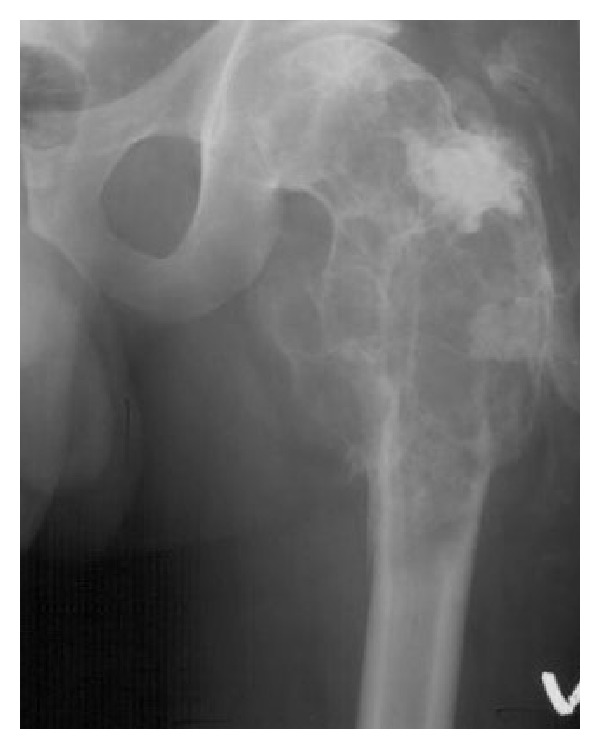
Radiograph from the original tumour.

**Figure 2:**
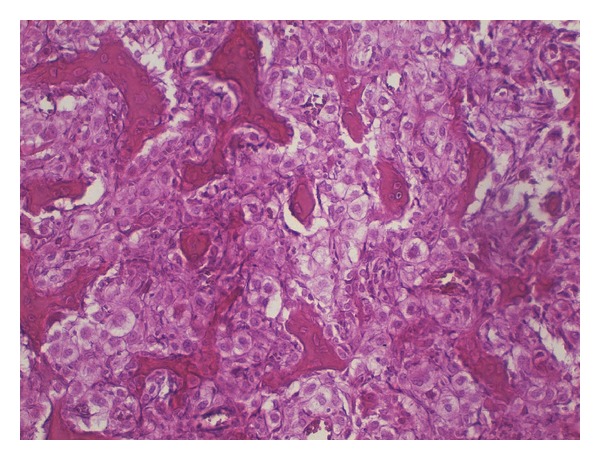
Typical area of clear cell chondrosarcoma diagnosed later from the original tumour specimen.

In 1982, a 33-year-old male patient presented with a pathological fracture of the left proximal femur. X-ray images of the proximal femur showed a large lytic lesion involving the whole intertrochanteric area. The lesion was expansive and bubby (Figure 1). An open biopsy was taken the day after the patient was admitted to hospital. The histology was difficult. Sheets of roundish cells with moderate nuclear pleomorphism were seen. Their cytoplasm was predominantly clear to granular (Figure 2). The lesion was initially mistaken for benign osteoblastoma. Since the lesion was diagnosed as benign no further examinations were undertaken. The fracture was supported with intramedullary nails until a custom-made endoprosthesis was available. Two months later the final intralesional resection was done and custom-made Link endoprosthesis was applied. Later the final pathology report from the resection was still difficult, but the treating physician concluded it might be an osteoblastoma or low-grade osteosarcoma. The surgery could be defined as intralesional and follow-up was conducted. The patient subsequently returned to near normal activities. He was working for 30 years with moderate physical strength but was unable to play any sport.

He was followed-up for ten years with local X-rays as well as lung X-rays and no evidence of local recurrence or distant metastasis was found. The latest X-ray from the operated femur was taken in 1993 and from the lung in 1994.

**Figure 3:**
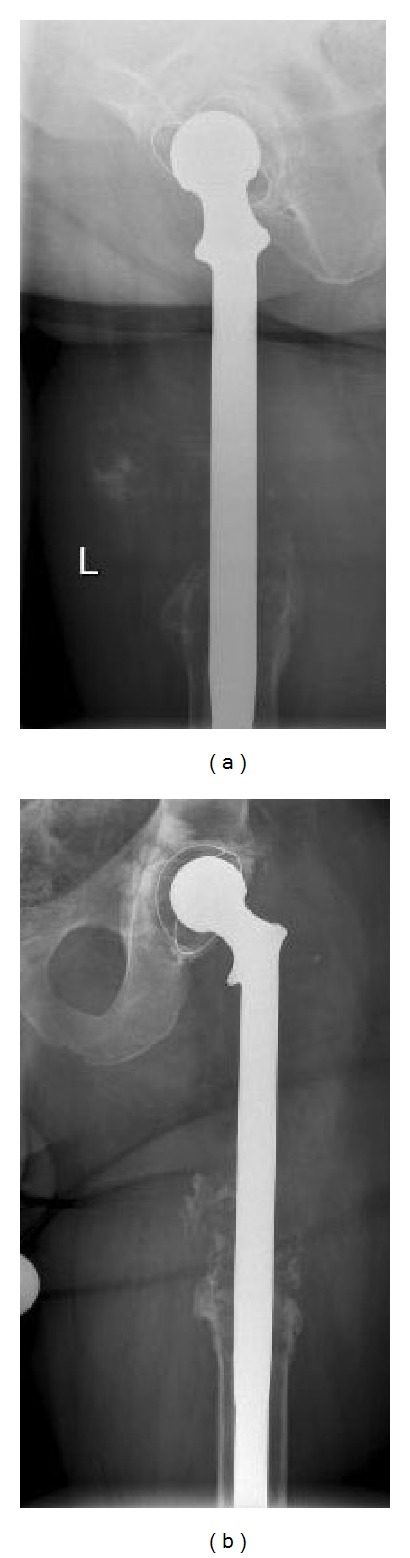
(a) Radiographs showing ectopic ossification on the lateral view revealing local recurrence. (b) AP view.

**Figure 4:**
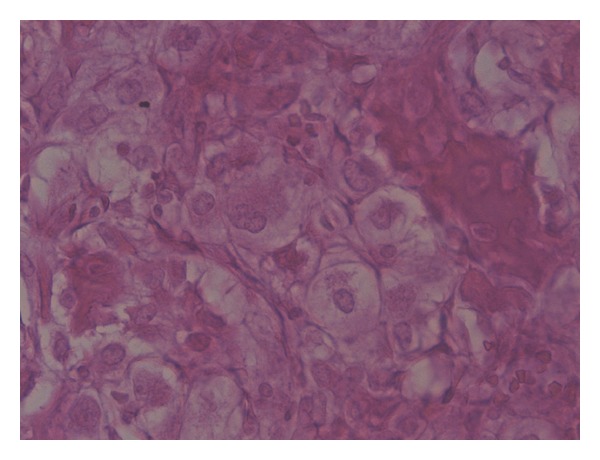
A view of clear cell chondrosarcoma showing large tumour cells, round to oval in shape, abundant clear cytoplasm and centrally located round nucleus.

In November 2011, 29 years after the primary surgery, the patient was referred to our institution because of symptoms suggestive of prosthesis loosening. He had symptoms such as pain and uncertainty in every step, but otherwise his overall health status was excellent. Because of locally increasing symptoms he had taken one cane for support, but otherwise he was able to walk unlimited distances. X-ray showed a typical aseptic loosening of the prosthesis with substantial subsidence. On the lateral X-ray some ectopic ossification could be observed at the prosthesis-bone junction (Figure 3). Magnetic resonance imaging (MRI) revealed a tumorous lesion anterior to the endoprosthesis in the prosthesis-bone junction. A Tru-Cut biopsy of the lesion was taken and this confirmed the CCCS. The original resected specimens were compared to this new lesion and histologically the primary tumour resected 29 years previously was identical to the new biopsy (Figure 4). Both the open biopsy sample and the resected tumour specimen from 1982 were now diagnosed as a CCCS. All lesions were characterized by the presence of clear cells arranged in a microlobular pattern. The constant feature was the presence of large tumour cells, round to oval in shape, with abundant clear cytoplasm and centrally located round nucleus. Further staging studies were done and surprisingly in lung computerized tomography (CT) multiple large tumours and smaller nodules were found. The biggest tumour was more than 7 cm in diameter and all together 20 nodules were found bilaterally. A biopsy of one lung nodule was taken to confirm the origin of the lung metastasis to be CCCS. Some of the lung metastases were located so medially that it was impossible to resect all metastases without a substantial risk of major complications.

**Figure 5:**
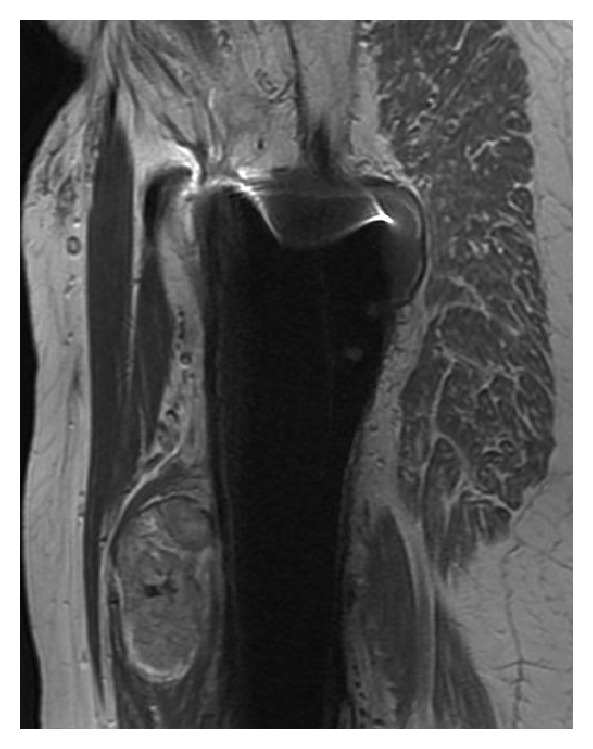
Sagittal T1-weighted MR image. The lesion is well defined at the prosthesis bone junction.

The local recurrence located in soft tissue area adjacent to the prosthesis-bone junction (Figure 5). Bony structures: distal femur and acetabulum showed no signs of tumour growth in MR images even though they both were reamed during the original intralesional procedure. Local recurrence together with the primary endoprosthesis was resected* en bloc* with wide margins. The recurrent tumour was solitary and well circumscribed and there were no satellite tumour clusters to be found. Tissue samples were taken intramedullarily from distal femur as well as from acetabulum and they both revealed to be free from tumour. The reconstruction was made with new modular tumour prosthesis. No further treatment was given.

**Figure 6:**
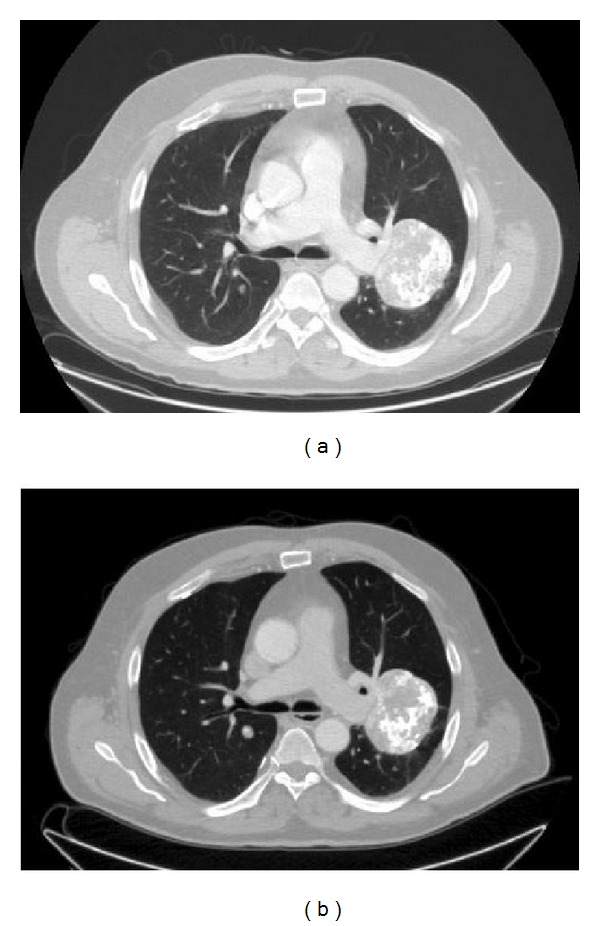
(a) Primary CT from the lung. (b) Control CT from the lung after two and half years of follow-up.

Two and a half years later there is no recurrence in the femur and all nodules in lungs have remained exactly the same in size and number (Figures 6(a) and 6(b)). The patient has continued to do well and he moves unrestrictedly.

## 3. Discussion

**Figure 7:**
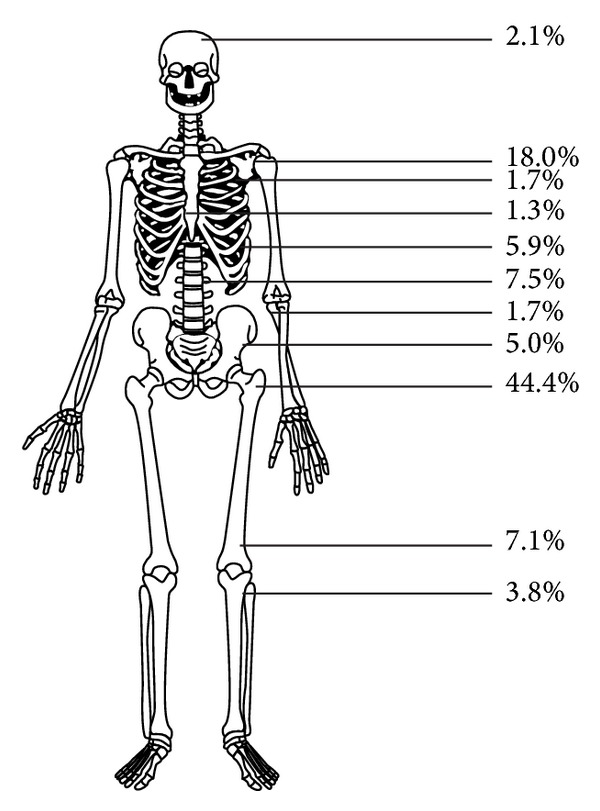
Anatomic distribution of 239 clear cell chondrosarcomas reported in 44 articles.

Clear cell chondrosarcoma is known to be a low-grade malignant tumour. To date, about 200 cases with clinical data have been reported in the literature. The prediagnosed symptoms are known to last long and delay to definitive diagnosis has been reported to vary from a few months to many years [1, 2, 6, 33, 41]. The femoral head has been the most typical site followed by numerous other locations. We collected information on the anatomic distribution from 44 articles and 239 cases and found that, as is known, the most frequent site for the tumour is the proximal femur (in 44.4%), followed by the proximal humerus (18.0%), the spine (7.5%), the distal femur (7.1%), the rib (5.9%), and the pelvis (5.0%) (Figure 7).

The rarity and slow growing potential of this tumour quite often leads to prolonged symptoms and also to initial misdiagnosis. The proportion of intralesional surgery has diminished over time, since awareness of this tumour has increased. However, the number of intralesional surgeries is still quite high and leads to a huge risk of local recurrence. The risk of recurrence varies between 40% and 100%, increasing with prolonged follow-up. The risk of local recurrence is markedly decreased by wide resection, but recurrences are still frequent with reported rates between 0 and 33%. Local recurrence may be extremely late, no matter whether treated intralesionally or with wide resection [1–3, 6, 41]. Local recurrences have been reported 24 years after the original diagnosis [41] and skeletal metastases 23 years after initial diagnosis [33]. Our case even as an extreme example of late recurrence and metastasis is in accordance with the literature, where late occurrence of local recurrence and metastases is frequent and therefore recommendations that such patients should be followed-up for prolonged periods or even lifelong are not exaggerated.

The progression of the disease seems to be slow even after local recurrence. Multiple resections due to numerous recurrences can be done and survival may still be many years or even decades [3, 6, 23, 39, 41]. Therefore an aggressive approach seems to be justified in local recurrences. CCCS metastases have two preferred sites, the bony skeleton and the lungs. Metastases may occur soon after diagnosis but late occurrences have also been reported. Most typically metastases are seen after the first or multiple local recurrences but they can occur with no previous history of recurrences especially in cases of wide resection [1, 2, 41, 42]. The majority of metastases are seen in bones or lungs, but some cases of soft tissue and brain metastases have been reported [3, 41]. Disease progression especially after bone metastasis may be surprisingly slow. Reports of surgically resected lung metastases are not found in the literature, but aggressive treatment of bone metastases has frequently been reported [1–3, 5, 6, 10, 41, 42]. Somehow, the slow clinical progression of even lung metastasis could lead to the consideration of more aggressive treatment. After two and half years of follow-up, lung CT revealed no increase in the size or number of lung metastases in our patient. The physical health is still unimpaired. This raises questions about systemic treatment. Chemotherapy or radiation therapy in the treatment of CCCS is controversial. Chondrosarcomas are overall poorly responsive to chemotherapy and radiation, and the literature does not support the use of them in oncological treatment. Some cases have been treated with radiation therapy, with or without success [2, 3, 41]. Chemotherapy was never used successfully in the reported cases.

## 4. Conclusion

CCCS is a low-grade tumour with greatly elevated risk of recurrence after intralesional surgery and therefore wide resection is the suggested adequate surgical method. Metastases mostly occur in bony skeleton or lungs and skeletal metastases seem to be less aggressive in the clinical course. Local recurrence and metastases may also occur after an extended period of time and follow-up is necessary for decades or lifelong. Aggressive treatment of local recurrences and possibly also metastases could be acceptable.

## Figures and Tables

**Table 1:** Summary of published studies on clear cell chondrosarcomas with more than five patients and clinical data.

Study	Patients	Intralesionally resected/local recurrence	Time of recurrence	Wide resection/local recurrence	Time of recurrence	Time of metastasis (range)	Number of lethal cases	Location of metastasis	Longest survival after recurrence	Longest survival after metastasis	Longest time to recurrence	Longest time to metastasis
Unni et al. 1976 [3]	16	44%/86%	2.1 y (0.3 y–5 y)	54%/none	—	1 y (1 y–1.2 y)	5/16	Lung Bone Brain	7.3 y	1.2 y	5 y	1.2 y

Le Charpentier et al. 1979 [5]	5	20%/100%	3 y	80%/none	—	2 y	1/5	Lung	10 y	2 y	3 y	2 y

Bjornsson et al. 1984 [2]	47	32%/86%	Range 0.2 y–5 y	52%/16%	4 y (2 y–6 y)	Range 2 y–12 y	7/47	Lung Bone	13 y	6 y	6 y	12 y

Ayoub et al. 1999 [6]	6	50%/66%	3.9 y (2.8 y–5 y)	50%/33%	19 y					4.2 y	19 y	1 y

Itälä et al. 2005 [1]	16	44%/71%	1.7 y (0.1 y–1.9 y)	56%/none	—	8.1 y (4–16.4 y)	1/16	Lung Bone	5-year survival 50%	8.4 y	1.9 y	16.4 y

Corradi et al. 2006 [42]	6	33%/100%	1.2 y (0.3 y–2 y)	67%/none	—	2.5 y (0.6 y–5 y)	0/6	Bone	5.4 y	4.8 y	7.5 y	9.7 y

Donati et al. 2008 [41]	18	28%/40%	4.4 y (1.0 y–11 y)	72%/17%	Range 2.2 y–10 y	Range 1 y–11 y	0/18	Bone Soft tissue		5 y	11 y	10 y
